# Chemically Binding Scaffolded Anodes with 3D Graphene Architectures Realizing Fast and Stable Lithium Storage

**DOI:** 10.34133/2019/8393085

**Published:** 2019-08-19

**Authors:** Ping Wu, Zhiwei Fang, Anping Zhang, Xiao Zhang, Yawen Tang, Yiming Zhou, Guihua Yu

**Affiliations:** ^1^Materials Science and Engineering Program and Department of Mechanical Engineering, The University of Texas at Austin, Austin, Texas 78712, USA; ^2^Jiangsu Key Laboratory of New Power Batteries, Jiangsu Collaborative Innovation Center of Biomedical Functional Materials, School of Chemistry and Materials Science, Nanjing Normal University, Nanjing 210023, China

## Abstract

Three-dimensional (3D) graphene has emerged as an ideal platform to hybridize with electrochemically active materials for improved performances. However, for lithium storage, current anodic guests often exist in the form of nanoparticles, physically attached to graphene hosts, and therefore tend to detach from graphene matrices and aggregate into large congeries, causing considerable capacity fading upon repeated cycling. Herein, we develop a facile double-network hydrogel-enabled methodology for chemically binding anodic scaffolds with 3D graphene architectures. Taking tin-based alloy anodes as an example, the double-network hydrogel, containing interpenetrated cyano-bridged coordination polymer hydrogel and graphene oxide hydrogel, is directly converted to a physical-intertwined and chemical-bonded Sn−Ni alloy scaffold and graphene architecture (Sn−Ni/G) dual framework. The unique dual framework structure, with remarkable structural stability and charge-transport capability, enables the Sn−Ni/G anode to exhibit long-term cyclic life (701 mA h g^−1^ after 200 cycles at 0.1 A g^−1^) and high rate performance (497 and 390 mA h g^−1^ at 1 and 2 A g^−1^, respectively). This work provides a new perspective towards chemically binding scaffolded low-cost electrode and electrocatalyst materials with 3D graphene architectures for boosting energy storage and conversion.

## 1. Introduction

Graphene, a two-dimensional (2D) nanostructure of carbon, has received considerable attention in energy, environmental, biomedical, and nanoelectronic fields, owing to its large surface area, superior electrical conductivity, high mechanical strength, and so on [1]. For electrochemical energy-related applications, building 3D graphene materials from 2D units can effectively prevent the self-stacking and thus maintain the unique physicochemical properties of graphene sheets [2–4]. Moreover, 3D graphene architectures with continuous graphene network and interconnected porosity can offer robust mechanical stability and highly efficient mixed-transport pathway for both electron and ions during electrochemical applications [2–4]. Therefore, 3D graphene has emerged as an ideal platform to hybridize with electrochemically active materials for improved energy storage and electrocatalytic performances [5–7].

With respect to lithium storage, searching for alternative anodes to commercial graphite with limited capacity has become an urgent task, so as to meet the ever-growing requirements in high-energy Li-ion batteries (LIBs) [8, 9]. However, current high-capacity anodes, with alloying-type and conversion-type Li-storage mechanisms, suffer intrinsically from poor structural stability and unsatisfied charge-transport capability during lithium insertion/extraction [10, 11]. Integration of these high-capacity anodes with 3D graphene can improve their structural stability, to a large extent, since the alloying- and conversion-based Li-storage reactions are confined within graphene buffering matrices [3, 4]. Meanwhile, the continuous graphene skeleton together with interconnected channels accelerates electron transport and lithium-ion diffusion in the 3D hybrid anodes [3, 4]. So far, a series of metals, alloys, oxides, sulfides, and phosphides have been integrated with 3D graphene to achieve improved cycle life and enhanced rate capability toward lithium storage [12–17]. Nevertheless, these anodic guests often exist in the form of nanoparticles, physically attached to graphene hosts, and therefore tend to detach from graphene matrices and aggregate into large congeries, causing considerable capacity fading under repeated Li insertion/extraction. As compared with 0D nanomaterials, anisotropic nanostructures with higher dimensions especially 3D scaffolded architectures manifest collective advantages of both nano-building units and microsized assemblies and thus greatly enhanced structural stability and capacity retention [18–21]. Moreover, interface chemistry engineering plays an increasingly significant role in designing progressive energy materials, and chemically bonded hybrid anodes are able to exhibit further-enhanced lithium-storage kinetics and performance than the ones hybridized in physical level [22–27]. In this regards, exploring approaches for chemically binding anodic scaffolds with 3D graphene architectures is of great potential in developing advanced electrode materials for LIBs.

**Figure 1:**
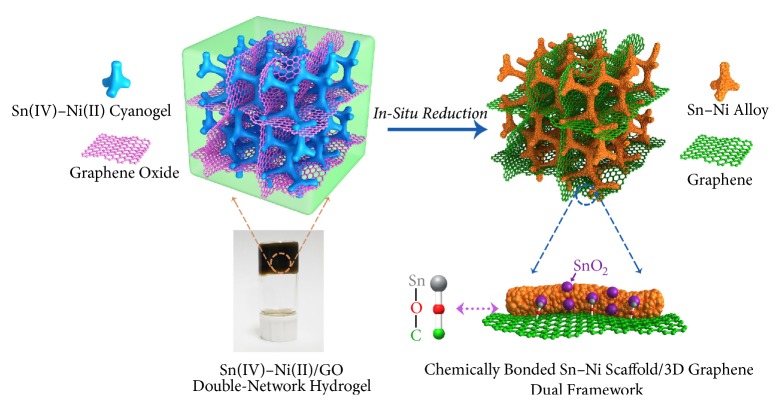
Synthetic and structural diagram of the chemically bonded Sn–Ni scaffold/3D graphene dual framework.

Herein, we develop a facile hydrogel-reduction route for chemically binding scaffolded anodes with 3D graphene architectures using double-network hydrogels as the precursors. Taking tin-based alloy anodes as an example, cyano-bridged Sn(IV)−Ni(II) coordination polymer hydrogel (Sn(IV)−Ni(II) cyanogel) and graphene oxide (GO) hydrogel have been selected to build Sn(IV)−Ni(II)/GO double-network hydrogel. As illustrated in Figure 1, the highly interpenetrated cyanogel and GO gel networks are concurrently reduced, and as a result, the obtained Sn−Ni alloy scaffold and graphene architecture are physically intertwined and chemically bonded* via* Sn−O−C bonds, yielding the final Sn−Ni/G dual framework. When applied as an anode for LIBs, the Sn−Ni/G dual framework manifests markedly enhanced lithium-storage performance in terms of reversible capacity, cycle life, and rate capability compared with single Sn−Ni scaffold.

## 2. Results and Discussion

The metal-containing part of the double-network hydrogel,* i.e.*, Sn(IV)−Ni(II) cyanogel ([Supplementary-material supplementary-material-1]), can be obtained through simply mixing aqueous solutions of SnCl_4_ and K_2_Ni(CN)_4_. After mixing, the nitrogen end from cyano ligand coordinates with Sn(IV) center, forming bridges between Ni(II) and Sn(IV) centers (Ni–C≡N–Sn), and this coordination reaction generates the extended cyano bridges and final cyanogels [21, 28–33]. For the GO part, polyvinyl pyrrolidone (PVP), a hydrogen-bond acceptor, acts as an efficient cross-linker for the gelation of GO, and the hydrogen-bond interaction between PVP chains and GO sheets can be responsible for the formation of the GO hydrogel, as revealed in [Supplementary-material supplementary-material-1] [34, 35]. Thus, the Sn(IV)–Ni(II)/GO double-network hydrogel ([Supplementary-material supplementary-material-1]) can be obtained through simultaneous coordination reaction between SnCl_4_ and K_2_Ni(CN)_4_ and hydrogen-bond interaction between PVP and GO. As a result, Sn(IV)–Ni(II) cyanogel and GO gel networks are highly interpenetrated in the double-network hydrogel.

**Figure 2:**
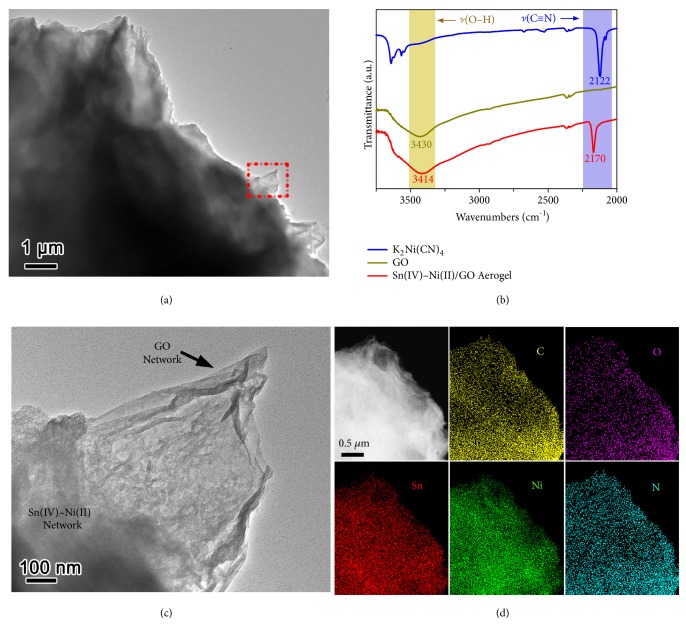
(a, c) TEM images, (b) FTIR spectrum, and (d) STEM-EDX elemental mappings of the Sn(IV)–Ni(II)/GO double-network aerogel.

To confirm the formation of the double-network gel, the structural and compositional features of the Sn(IV)–Ni(II)/GO aerogel have been examined (Figure 2). Figure 2(b) displays the Fourier transform infrared (FTIR) spectrum of the Sn(IV)–Ni(II)/GO aerogel in comparison with K_2_Ni(CN)_4_ reagent and GO sheets. As can be seen, the FTIR spectrum of the double-network aerogel reveals a shift of cyano stretching vibration to a higher frequency at 2170 cm^−1^ compared with K_2_Ni(CN)_4_ reagent (2122 cm^−1^). Such positive shift of *ν*(C≡N) indicates the formation of the structural unit of Sn(IV)−Ni(II) cyanogel (Ni–C≡N–Sn), in analogy to similar bridging cyano groups including Ni–C≡N–Sb [28], Fe–C≡N–Sn [29–31], and Co–C≡N–In [32] in other cyanogel systems. Moreover, a negative shift of *ν*(O–H) from 3430 cm^−1^ in GO sheets to 3414 cm^−1^ in the double-network aerogel is clearly observed. This shift of hydroxyl stretching vibration is generally characteristic of hydrogen-bond interaction, suggesting the presence of hydrogen bonds between PVP chains and GO sheets [34, 35]. The FTIR results verify the formation of both cyanogel and GO gel networks in the Sn(IV)–Ni(II)/GO double-network gel.

Figures 2(a) and 2(c) reveal the transmission electron microscopy (TEM) image of the Sn(IV)–Ni(II)/GO aerogel. As observed, the aerogel product possesses the mutual structure features of gel-based materials and manifests a 3D framework structure [36–38]. More specifically, Sn(IV)–Ni(II) cyanogel network is effectively and uniformly integrated into GO gel network in the double-network gel, by analogy to single-network cyanogel and GO gel ([Supplementary-material supplementary-material-1]). The simultaneous gelation reactions (coordination reaction and hydrogen-bond interaction) for cyanogel and GO gel guarantee the formation of the integrative double-network gel, which can be further confirmed by its scanning transmission electron microscopy (STEM)-energy-dispersive X-ray spectrometer (EDX) elemental mappings (Figure 2(d)). The observed element signals of Sn, Ni, and N come from the cyanogel network, and O elemental signal originates from GO gel network, whereas carbon signal is contributed from these two networks. The homogeneous distribution of these elemental signals within the entire 3D framework reveals that the Sn(IV)–Ni(II) cyanogel and GO gel networks are highly interpenetrated in the double-network gel.

**Figure 3:**
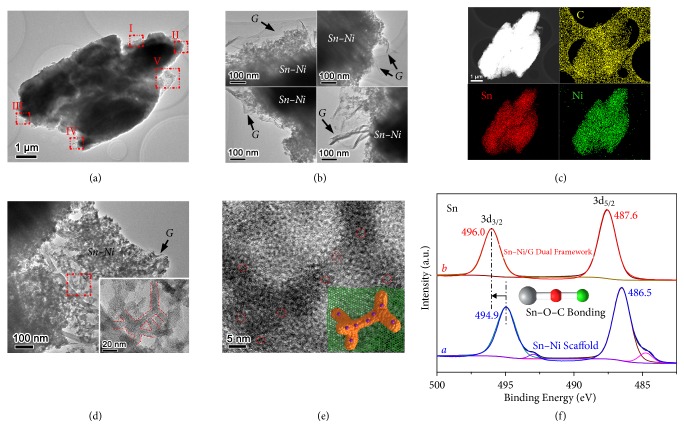
(a,b,d) TEM images, (c) STEM-EDX elemental mappings, (e) HRTEM image, and (f) Sn 3d XPS spectrum of the Sn–Ni/G dual framework.

In this gel precursor route, the formation of integrative double-network gel is a prerequisite of subsequently incorporating scaffolded Sn−Ni alloy anode into 3D graphene architectures for boosting lithium storage. After an aqueous sodium borohydride reduction process, the Sn(IV)–Ni(II) cyanogel and GO gel networks are directly transformed into scaffolded Sn−Ni alloy and graphene architecture, respectively, yielding the final Sn−Ni/G dual framework.  Figure 3(a) reveals the TEM image of the Sn−Ni/G framework. As clearly seen, this Sn−Ni/G product inherits the structural features of Sn(IV)–Ni(II)/GO gel and exists in the form 3D nanoporous framework. The nanoporous feature of this framework has been further revealed by N_2_ adsorption/desorption test ([Supplementary-material supplementary-material-1]), and the high surface area (159.3 m^2^ g^−1^) and large pore volume (0.49 cm^3^ g^−1^) with average pore size of 14.9 nm facilitate the electrode-electrolyte contact and stress release of this framework anode upon lithium insertion/extraction [28–33, 36–38]. The magnified views corresponding to regions I to IV in Figure 3(a) clearly reveal the coexistence of Sn−Ni scaffold and graphene architecture in all the edge areas (Figure 3(b)), indicating that the alloy and graphene components are highly intertwined in the dual framework. Moreover, the STEM-EDX elemental mappings demonstrate the homogeneous distribution of Sn, Ni, and C elemental signals within the entire framework, further confirming the uniform incorporation of Sn−Ni alloy scaffold into 3D graphene architecture (Figure 3(c)). Additionally, the graphene content is determined to be ~27.9 wt% in the hybrid framework ([Supplementary-material supplementary-material-1]). For comparison, Sn−Ni alloy control sample has been prepared by reducing Sn(IV)−Ni(II) cyanogel instead of the double-network hydrogel and exists in the form of single alloy scaffold with uniformly distributed Sn and Ni elements ([Supplementary-material supplementary-material-1]).

To gain deeper insight into the Sn−Ni/G dual framework material, the microscopic structural features of an edge part containing exposed graphene and broken alloy scaffold have been further examined (Figures 3(d) and 3(e)). As can be seen, scaffolded Sn−Ni alloy is effectively incorporated into 3D graphene network (Figure 3(d)). The magnified TEM image clearly reveals that the alloy scaffold is assembled by 1D nanodendrites with an average diameter of about 10 nm, as highlighted by red dotted lines, and the terminal nanodendrites of the scaffold are firmly attached to graphene surface (Inset in Figure 3(d)). With respect to crystalline state, Sn−Ni alloy is amorphous in nature, as confirmed by its X-ray powder diffraction (XRD) pattern ([Supplementary-material supplementary-material-1]) and high-resolution TEM (HRTEM) image (Figure 3(e)). Additionally, the observed ultrasmall nanocrystals with a crystalline size of 2~3 nm from HRTEM image originate from the native tin oxide anchored on Sn–Ni alloy surface. Amorphous alloys possess isotropic volumetric expansion and good tolerance to intrinsic strain/stress and thus are capable of extended cycling toward lithium storage [39, 40]. Meanwhile, the oxide crystals might be beneficial to the effective attachment between the alloy scaffold and graphene matrix* via* interface chemical interactions, which can be confirmed by X-ray photoelectron spectroscopy (XPS) analysis. The atomic ratio of tin and nickel is determined to be 1.1:1 by XPS survey spectrum in the dual framework, very close to the feeding ratio of SnCl_4_ and K_2_Ni(CN)_4_ reactants ([Supplementary-material supplementary-material-1]).  Figure 3(f) displays the Sn 3d XPS spectrum of the Sn–Ni/G dual framework in comparison with single Sn–Ni scaffold. For dual framework, the peaks located at 496.0 and 487.6 eV can be assigned to Sn 3d_3/2_ and 3d_5/2_, respectively (curve* b*), which are over 1.1 eV higher than those of Sn–Ni scaffold (curve* a*). The increased binding energy indicates a decreased electron density at Sn sites, and the positive shift can be attributed to the formation of Sn−O−C bonds between Sn–Ni alloy and graphene matrix since carbon has a higher electronegativity than that of tin [25]. The presence of Sn−O−C bonds is also confirmed by the O 1s and C 1s XPS spectra of the Sn–Ni/G dual framework ([Supplementary-material supplementary-material-1]) [26]. The strong interfacial Sn–O–C bonding not only prevents the detachment and aggregation of Sn–Ni alloy, but also accelerates charge transport between alloy scaffold and graphene matrix upon cycling [22–27].

**Figure 4:**
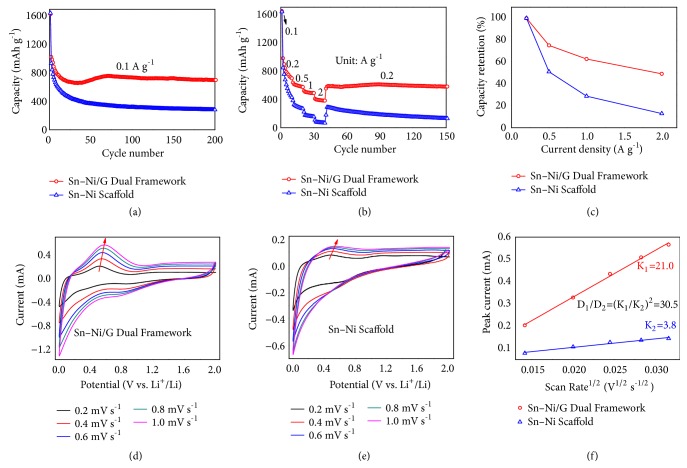
Lithium storage performance of the Sn–Ni/G dual framework in comparison with the Sn–Ni scaffold: (a) cycling stability, (b) rate capability, (c) rate retention, (d, e) CV curves at different scan rates, and (f) relationship between Ip and (V/s)^1/2^.

As a proof-of-concept illustration of the structural superiorities for dual framework anodes, we took Sn−Ni/G dual framework as a representative example and examined its cycle life and rate performance toward lithium storage.  Figure 4(a) displays the discharge capacity versus cycle number for the Sn−Ni/G dual framework compared with Sn−Ni scaffold (0.01-2 V, 0.1 A g^−1^). As shown, the Sn−Ni/G product delivers initial and second discharge capacities of 1630 and 1027 mA h g^−1^, respectively. The initial capacity loss can be mainly assigned to the irreversible formation of solid electrolyte interface (SEI) layer on anodic surface and could be mitigated or eliminated* via* prelithiation routes for its practical applications [41, 42]. Additionally, the discharge capacity slowly descends in the initial 30 cycles, increases gradually from 30 to 75 cycles, and tends to be stable in subsequent cycles. Similar capacity-rise phenomenon has also been observed from other tin-based alloy anodes and can be normally attributed to the electrochemical activation of these high-capacity anodes as well as the formation and stabilization of SEI components [43, 44]. Thus, the average capacity fading for the Sn−Ni/G dual framework is only 0.16% per cycle from 2 to 200 cycles, and this dual framework anode is able to deliver a high reversible capacity of 701 mA h g^−1^ in the 200^th^ cycle, much higher than the theoretical capacity of commercial graphite (372 mA h g^−1^). In sharp contrast, the discharge capacity of Sn−Ni scaffold decreases much faster during the entire cycling, and its average capacity fading reaches up to 0.35% per cycle from 2 to 200 cycles. Confining alloying-based Li-storage reactions within 3D graphene architectures can effectively accommodate volume variations and suppress electrode pulverization [12–17], and meanwhile, chemically bonded alloy scaffolds show better tolerance to mechanical strain/stress and thus enhanced structural stability compared to physical-attached nanoparticle counterparts [19–27], leading to the long-term cyclic life of the Sn−Ni/G dual framework.

Moreover, the interconnected 1D to 3D alloy platform and continuous graphene skeleton together with strong interfacial Sn–O–C bonding provide fast pathways for electrons [12–17, 22–27], and the internally abundant nanopores promote the contact between the electrolyte and dual framework electrode [19–21, 36–38]. Thanks to the unique mixed conducting networks for both electron and Li-ion, the Sn−Ni/G dual framework exhibits greatly enhanced charge-transport and high-rate capabilities. As shown in Figure 4(b), the specific capacities of the Sn−Ni/G product are all much higher than those of Sn−Ni control sample from 0.2 to 2 A g^−1^, and the dual framework is able to exhibit high average capacities of 793, 596, 497, and 390 mA h g^−1^ at 0.2, 0.5, 1, and 2 A g^−1^, respectively. Also, the capacity retention at 2 A g^−1^* vs* 0.2 A g^−1^ reaches up to 49% for Sn−Ni/G dual framework (Figure 4(c)), much higher than that of Sn−Ni scaffold (13%).

**Figure 5:**
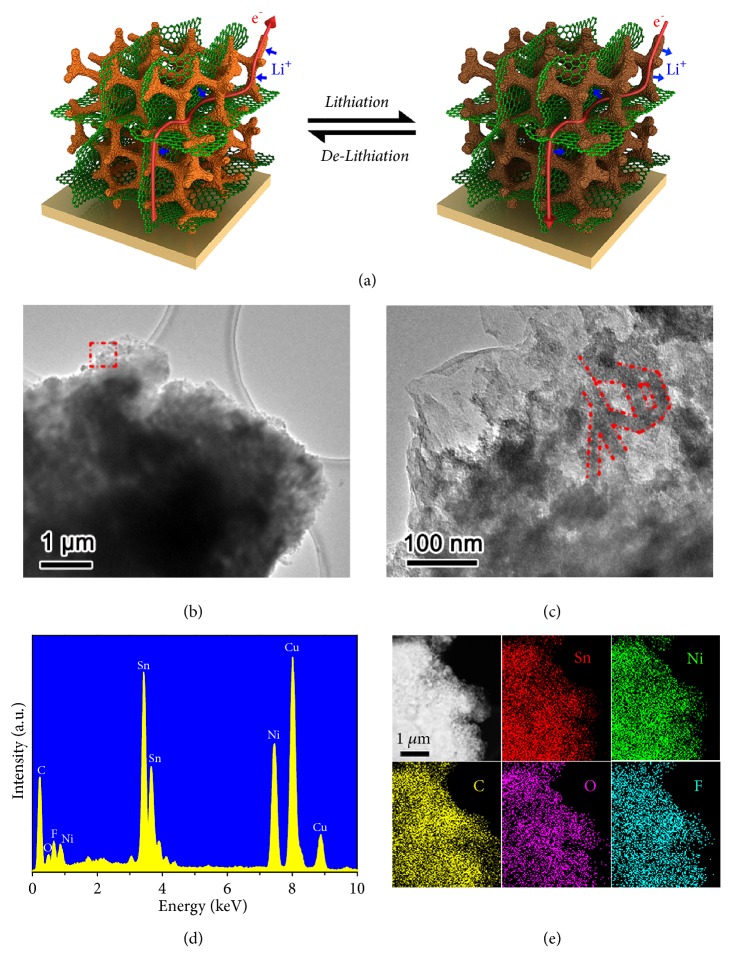
(a) Schematic illustration of the lithiation/delithiation processes for the Sn–Ni/G dual framework. (b, c) TEM images, (d) EDX spectrum, and (e) STEM-EDX elemental mappings of the Sn–Ni/G dual framework in a fully delithiated state (2 V* vs.* Li^+^/Li) after 200 cycles.

The cyclic life and rate performance of the Sn−Ni/G dual framework are comparable to those of state-of-the-art tin-based alloy anodes, as listed in [Supplementary-material supplementary-material-1]. The unique dual framework structure, containing physical-intertwined and chemical-bonded alloy scaffold and graphene architecture, is the key factor for the Sn−Ni/G hybrid electrode to achieve remarkable structural stability and charge-transport capability, which has been schematically illustrated by its lithiation/delithiation processes (Figure 5(a)) and further verified by electrochemical kinetics tests and structural characterization after cycling. Figures 4(d) and 4(e) show the cyclic voltammetry (CV) curves of the dual framework and Sn−Ni scaffold anodes at different scan rates (0.2 to 1.0 mV s^−1^). As observed, a characteristic pair of cathodic and anodic peaks at the potential of 0.0-0.6 V and 0.2-1.0 V can be assigned to the Li–Sn alloying and dealloying reactions (Sn + 4.4Li^+^ + 4.4e^−^*↔* Li_4.4_Sn) [29, 43]. Compared with Sn−Ni scaffold, the Sn–Ni/G dual framework shows lower electrochemical polarization and smoother lithium insertion/extraction reactions [43]. Specifically, the apparent diffusion coefficient of lithium ions (*D*_Li_^+^) can be calculated according to the following Randles-Sevcik equation [45]: *I*_p_ = 2.69 × 10^5^ n^3/2^ A *C*_o_*D*^1/2^*v*^1/2^, where *I*_p_ is the peak current, n is the charge-transfer number, A stands for the electrode surface area, *C*_o_ is the concentration,* D* is the Li-ion diffusion coefficient, and* v* is the scan rate. The peak currents from CV curves have been chosen for a liner fit (Figure 4(f)). Considering the similar alloying-type lithium-storage behavior and the same testing conditions, the values of n, A, and *C*_o_ should be consistent for these two samples. As a result, the Li^+^ diffusion coefficient of the Sn–Ni/G dual framework is more than 30 times higher than that of Sn–Ni scaffold. These results demonstrate that the charge-transport capability can be significantly improved through the chemical binding of scaffolded anodes with 3D graphene architectures, and the high Li^+^ diffusion coefficients ensure the superior rate performance of the dual framework anodes.

Figures 5(b)–5(e) display the microscopic structure of the Sn−Ni/G dual framework in a fully delithiated state after 200 cycles. As observed, the 3D nanoporous structure is well preserved, and the electrode agglomeration and pulverization phenomena can be effectively restricted upon repeated Li insertion/extraction (Figure 5(b)). The magnified view reveals that the delithiated Sn−Ni alloy also exists in the form of 1D to 3D scaffolded structure, and this alloy scaffold is still effectively integrated into graphene architecture after cycling (Figure 5(c)). Additionally, the elemental peaks of Sn, Ni, and C in EDX spectrum are expected from the dual framework, whereas O and F peaks originate from SEI layer containing Li_2_CO_3_, LiF, and so on (Figure 5(d)). Moreover, the STEM-EDX elemental maps demonstrate the homogeneous distribution of these elemental signals within the Sn−Ni/G electrode, indicating that the alloy scaffold and graphene architecture are still highly intertwined in the delithiated Sn−Ni/G dual framework (Figure 5(e)). The above results further verify the greatly enhanced strain-accommodation capability and remarkable structural stability of the dual framework anodes, which plays a critical role in their improved lithium-storage performance especially long-term cyclic life.

## 3. Conclusion

To summarize, we develop a facile hydrogel-reduction route for chemically binding anodic scaffolds with 3D graphene architectures using Sn(IV)–Ni(II)/GO double-network hydrogels as precursors. The simultaneous gelation reactions for cyanogel and GO gel guarantee the formation of integrative double-network gels, which is a prerequisite of subsequently incorporating Sn−Ni alloy scaffold into 3D graphene architecture effectively and uniformly. The unique dual framework structure, consisting of physical-intertwined and chemical-bonded alloy scaffold and graphene architecture, is the key factor for the Sn−Ni/G hybrid anode to achieve remarkable structural stability and charge-transport capability and thus realizing long-term cyclic life and high rate performance toward lithium storage. Moreover, the double-network hydrogel-enabled route could be easily extended to chemically bind other scaffolded electrode and electrocatalyst materials with 3D graphene architectures for boosting electrochemical performances.

## 4. Materials and Methods

### 4.1. Synthesis of the Sn(IV)–Ni(II)/GO Double-Network Hydrogel

Solution A was aqueous solution containing 0.2 M SnCl_4_ and 10 mg mL^−1^ of GO. Solution B was aqueous solution containing 0.2 M K_2_Ni(CN)_4_ and 2 mg mL^−1^ of polyvinyl pyrrolidone (PVP). The Sn(IV)–Ni(II)/GO double-network hydrogel was conveniently synthesized by mixing solutions A and B in a volume ratio of 1:1. For comparison, Sn(IV)–Ni(II) cyanogel was obtained by mixing SnCl_4_ aqueous solution with K_2_Ni(CN)_4_ aqueous solution. And GO hydrogel was prepared by mixing GO aqueous solution with PVP aqueous solution.

### 4.2. Synthesis of the Sn–Ni/G Dual Framework

The Sn–Ni/G dual framework was synthesized through a facile hydrogel-reduction route using Sn(IV)–Ni(II)/GO double-network hydrogel as a precursor. Specifically, 1 M NaBH_4_ aqueous solution was added to the Sn(IV)–Ni(II)/GO double-network hydrogel, and the reaction system was allowed to stand for 1 h. The black product was washed and dried in a vacuum oven at 80°C, yielding the final Sn–Ni/G dual framework. For comparison, Sn–Ni scaffold was synthesized* via* similar NaBH_4_ reduction processes using Sn–Ni cyanogel as a precursor instead of double-network hydrogel.

### 4.3. Materials Characterization

The morphology, composition and structure of these products were examined by X-ray powder diffraction (XRD, Rigaku D/max 2500/PC), scanning electron microscopy (SEM, Hitachi S-5500), and high-resolution transmission electron microscopy (HRTEM, JEOL JEM-2010F, 200 kV) equipped with an energy-dispersive X-ray spectrometer (EDX, Thermo Fisher Scientific). The Fourier transform infrared (FTIR) spectra were recorded on a Bruker Tensor 27 spectrometer. Nitrogen adsorption/desorption measurements were examined at 77 K using a Micromeritics ASAP 2050 analyzer, and the surface area, pore volume, and pore size were calculated using Brunauer–Emmett–Teller (BET) and Barrett–Joyner–Halenda (BJH) methods, respectively. X-ray photoelectron spectroscopy (XPS) test was conducted on an ESCALAB 250Xi Spectrometer (Thermo Scientific). Thermogravimetric analysis (TGA) was performed using a thermal analyzer (NETZSCH STA) with a heating rate of 10°C min^−1^ in air.

### 4.4. Electrochemical Measurement

The working electrodes were prepared from copper foil current collectors, coated beforehand with slurries containing active material (e.g., Sn–Ni/G dual framework), conductive material (Super P carbon black), and binder (sodium carboxymethyl-cellulose, CMC) in a weight ratio of 70:15:15. Then, the obtained electrodes were dried at 120°C for 12 h in a vacuum oven. The counter electrode was lithium foil, and the electrolyte was 1 M LiPF_6_ in ethylene carbonate/dimethyl carbonate (EC/DMC, 1:1 in volume). Electrochemical tests were examined using 2025-type coin cells (can size: 20 mm in diameter and 2.5 mm in thickness), which were assembled in an argon-filled glove box (IL-2GB, Innovative Technology). Cycling tests of the assembled cells were measured on a battery tester (LANHE CT2001A) in the voltage range of 0.01-2 V at different current densities (0.1 A g^−1^ for the first cycle and 0.1 to 2 A g^−1^ in subsequent cycles in the cycling and rate tests), and cyclic voltammetry (CV) measurements were conducted on a CHI 660B electrochemical workstation in the potential window of 0-2 V at various scan rates (0.2 to 1.0 mV s^−1^).

## Data Availability

All data generated or analyzed during this study are included in this published article and its Supplementary Materials.

## Supplementary Materials

Supplementary Materials Figure S1: photographs of Sn(IV)–Ni(II) cyanogel (a), GO hydrogel (b), and Sn(IV)–Ni(II)/GO double-network hydrogel (c) and their corresponding models (insets). Figure S2: TEM images of the Sn(IV)–Ni(II) cyanogel (a) and GO aerogel (b). Figure S3: (a) nitrogen adsorption and desorption isotherms and (b) pore diameter distribution from desorption branch of the Sn–Ni/G dual framework. Figure S4: TGA curve of the Sn–Ni/G dual framework. Figure S5: (a, b) TEM images and (c) STEM-EDX elemental mappings of the Sn–Ni scaffold. Figure S6: XRD patterns of the Sn–Ni/G dual framework (curve a) and its annealing product (curve b) annealed at 500°C for 1 h under flowing N2. Figure S7: XPS spectrum of the Sn–Ni/G dual framework. Figure S8: O 1s and C 1s XPS spectra of the Sn–Ni/G dual framework. Table S1: Comparison of the lithium storage performance between the Sn–Ni/G dual framework and previous Sn–M alloy-based anodes.
