# Supplementary material for: Chemically Binding Scaffolded Anodes with 3D Graphene Architectures Realizing Fast and Stable Lithium Storage
Source: Research (Wash D C). 2019 Aug 19;2019:8393085. doi: 10.34133/2019/8393085 (PMC6750113; doi:10.34133/2019/8393085)
Supplement: Supplementary Materials — Figure S1: photographs of Sn(IV)–Ni(II) cyanogel (a), GO hydrogel (b), and Sn(IV)–Ni(II)/GO double-network hydrogel (c) and their corresponding models (insets). Figure S2: TEM images of the Sn(IV)–Ni(II) cyanogel (a) and GO aerogel (b). Figure S3: (a) nitrogen adsorption and desorption isotherms and (b) pore diameter distribution from desorption branch of the Sn–Ni/G dual framework. Figure S4: TGA curve of the Sn–Ni/G dual framework. Figure S5: (a, b) TEM images and (c) STEM-EDX elemental mappings of the Sn–Ni scaffold. Figure S6: XRD patterns of the Sn–Ni/G dual framework (curve a) and its annealing product (curve b) annealed at 500°C for 1 h under flowing N2. Figure S7: XPS spectrum of the Sn–Ni/G dual framework. Figure S8: O 1s and C 1s XPS spectra of the Sn–Ni/G dual framework. Table S1: Comparison of the lithium storage performance between the Sn–Ni/G dual framework and previous Sn–M alloy-based anodes. [file 8393085.f1.docx]

**Supplementary Materials**


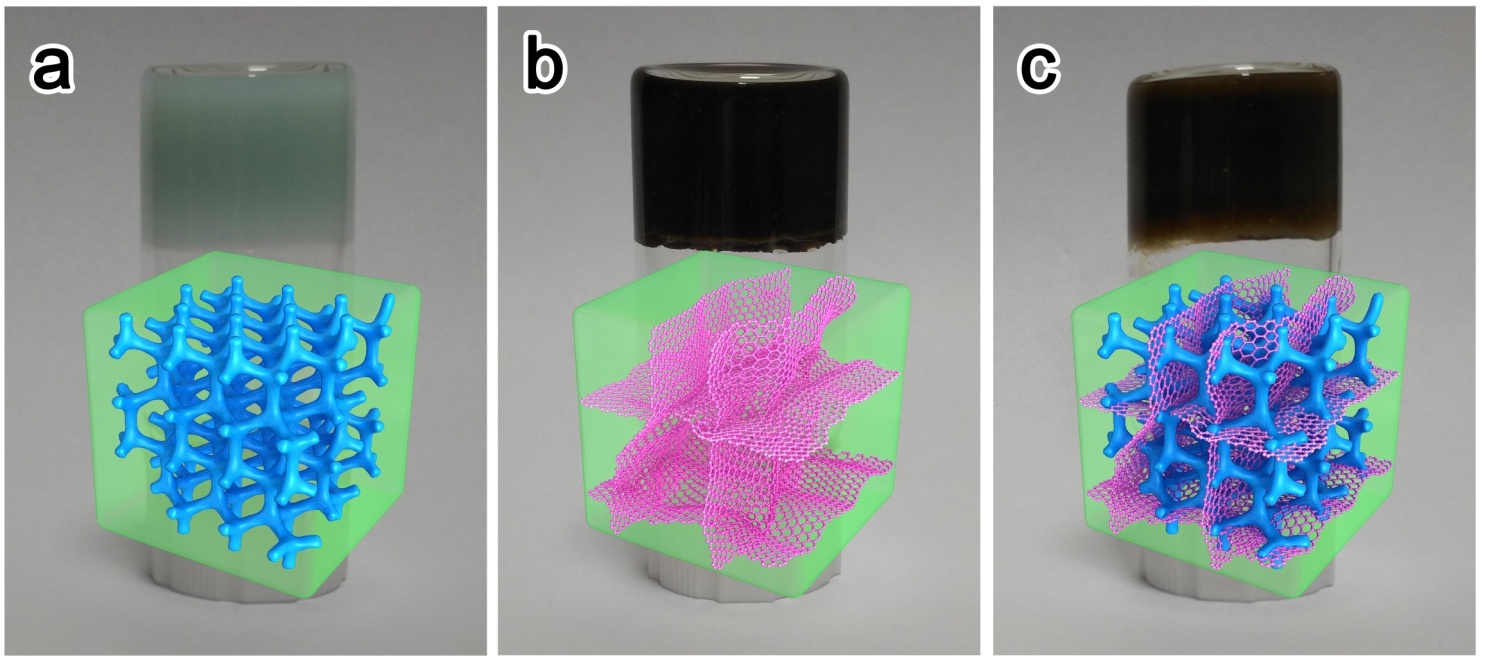


**Figure S1** Photographs of Sn(IV)–Ni(II) cyanogel (a), GO hydrogel (b), and Sn(IV)–Ni(II)/GO double-network hydrogel (c), and their corresponding models (insets).


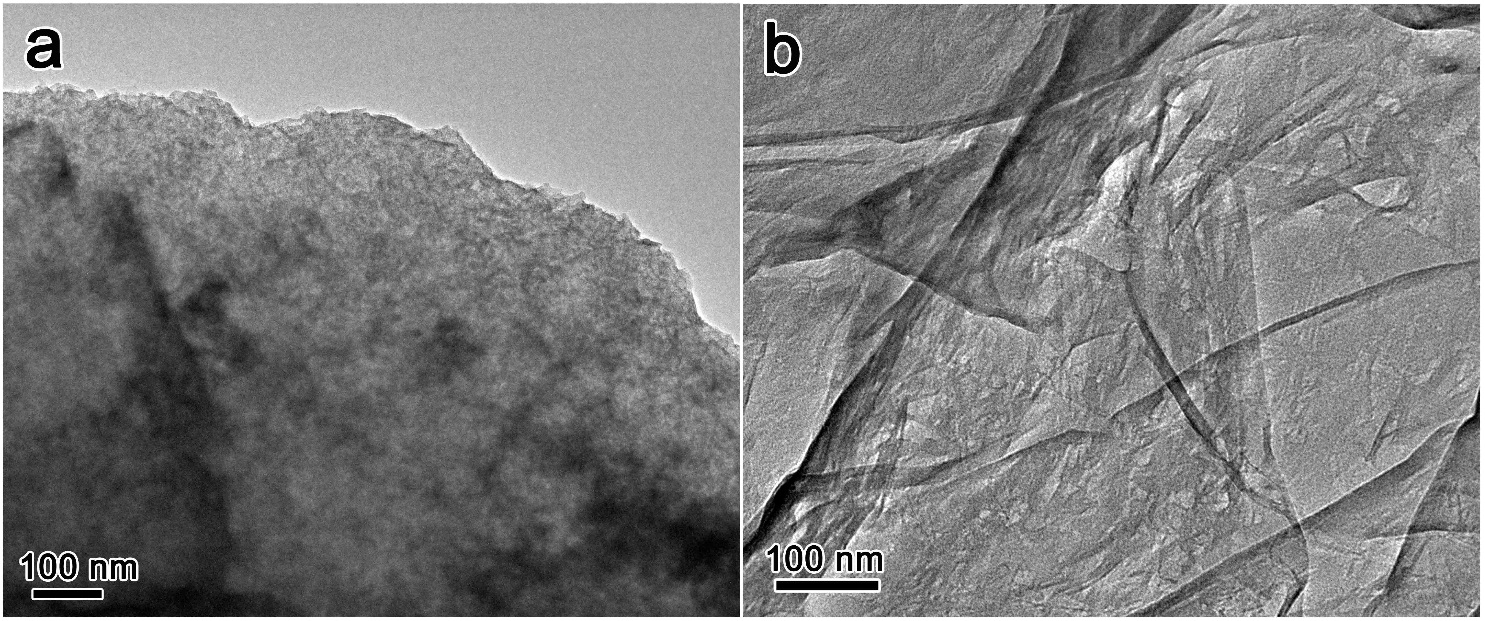


**Figure S2** TEM images of the Sn(IV)–Ni(II) cyanogel (a) and GO aerogel (b).


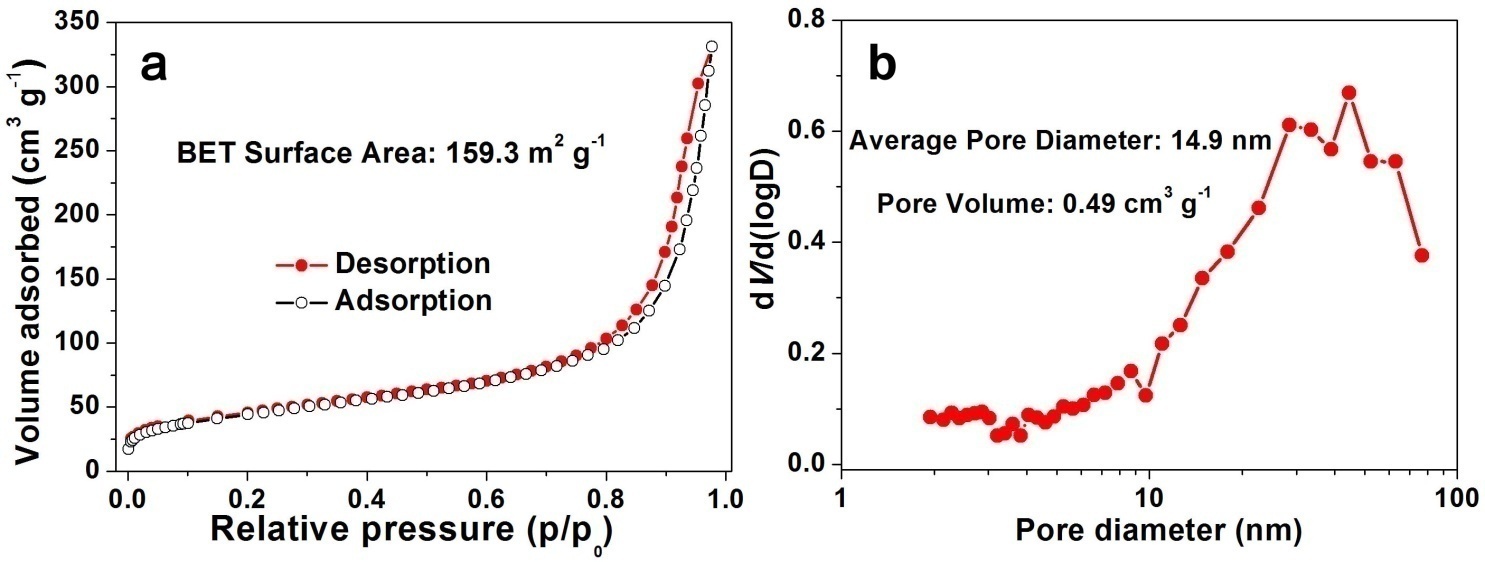


**Figure S3** (a) Nitrogen adsorption and desorption isotherms and (b) pore diameter distribution from desorption branch of the Sn–Ni/G dual framework.


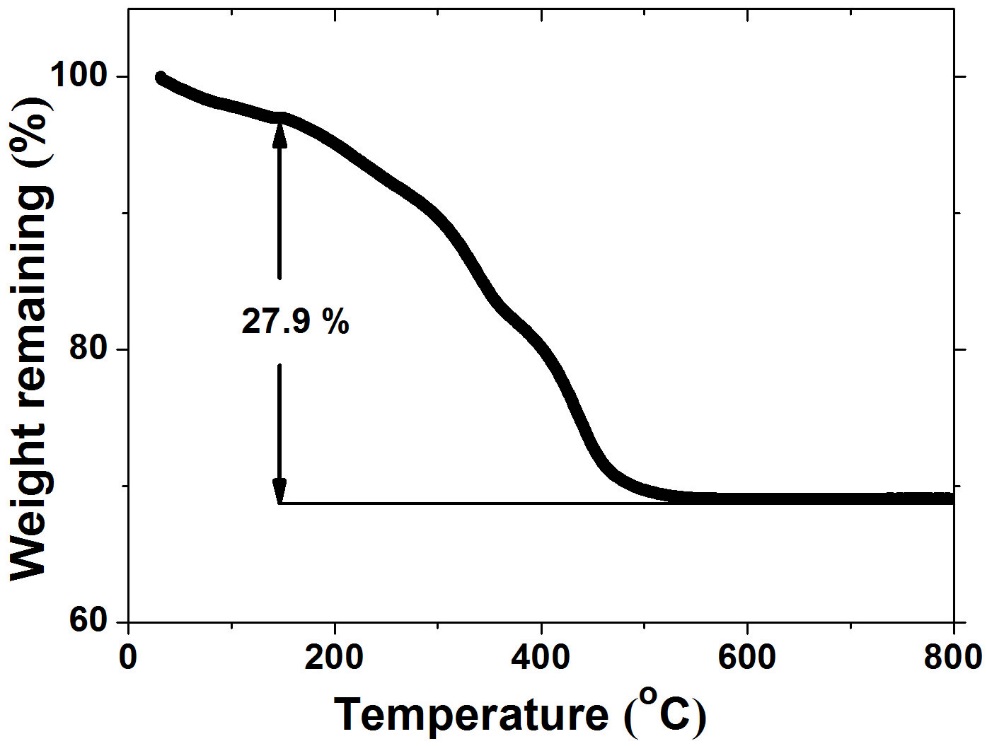


**Figure S4** TGA curve of the Sn–Ni/G dual framework.


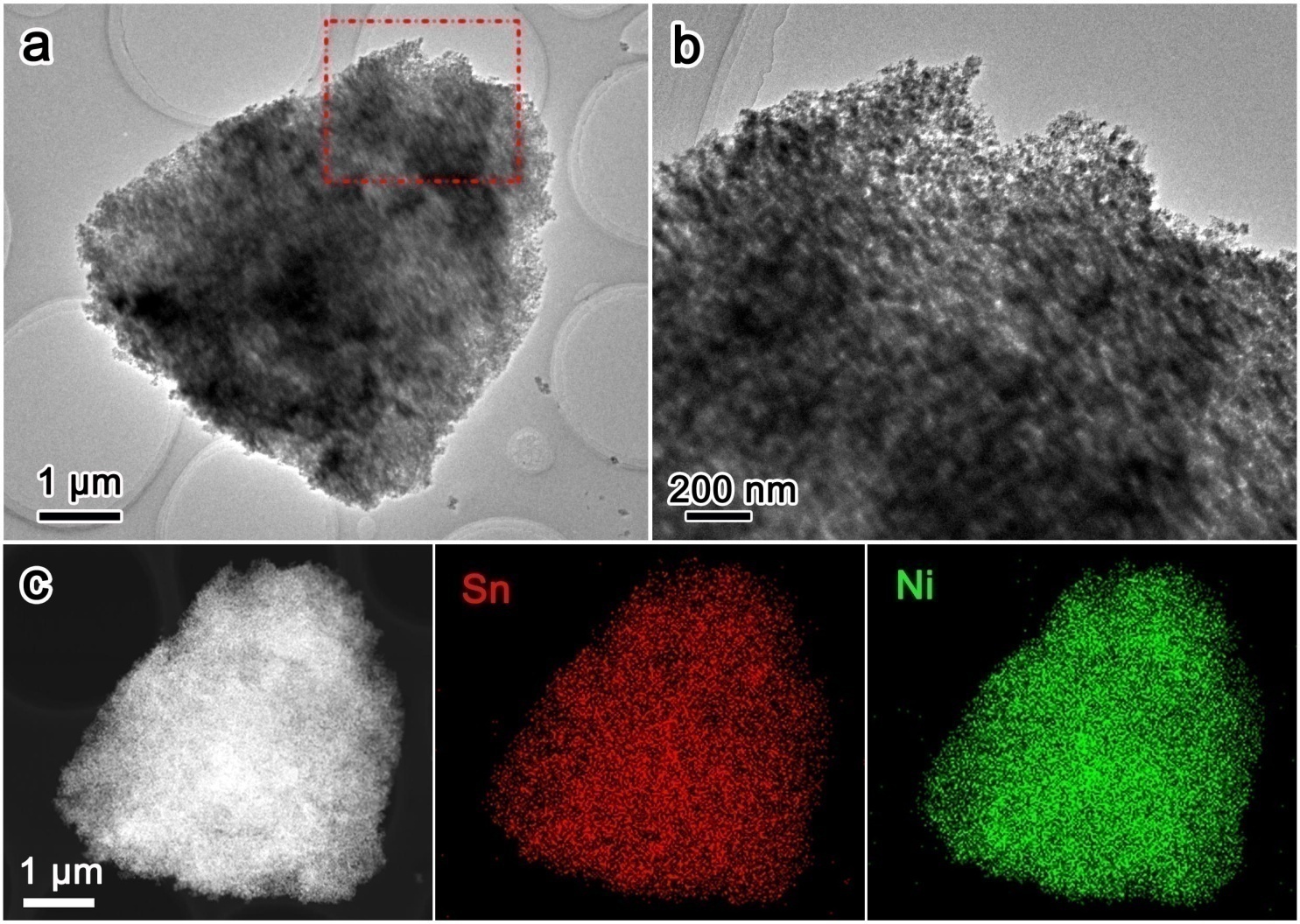


**Figure S5** (a, b) TEM images and (c) STEM-EDX elemental mappings of the Sn–Ni scaffold.


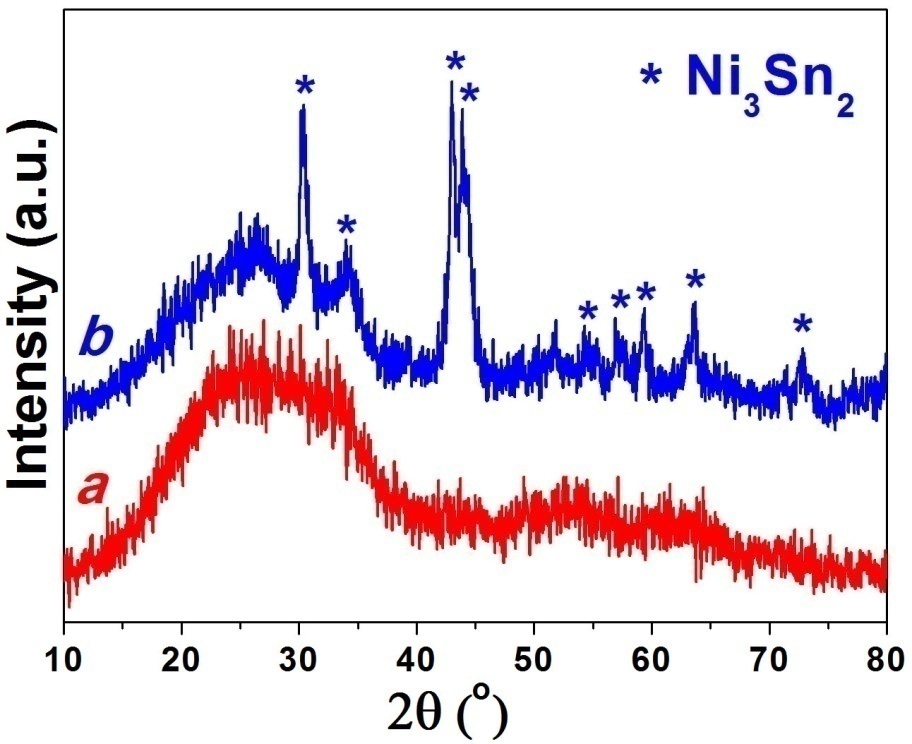


**Figure S6** XRD patterns of the Sn–Ni/G dual framework (curve *a*) and its annealing product (curve *b*) annealed at 500 ^o^C for 1 h under flowing N_2_.

Figure S6 shows the XRD patterns of the Sn–Ni/G dual framework (curve *a*) and its annealing product (curve *b*). As shown in curve *a*, Sn−Ni alloy in the Sn–Ni/G dual framework is amorphous in nature. To confirm the presence of Sn−Ni alloy, the Sn–Ni/G dual framework was annealed under flowing N_2_ at 500 ^o^C for 1 h, and the crystalline state of the annealing product was examined. The crystalline phase of orthorhombic Ni_3_Sn_2_ (JCPDS: 65-9650) can be clearly observed from curve *b*, further confirming the existence form of Sn–Ni alloy in the Sn–Ni/G dual framework.


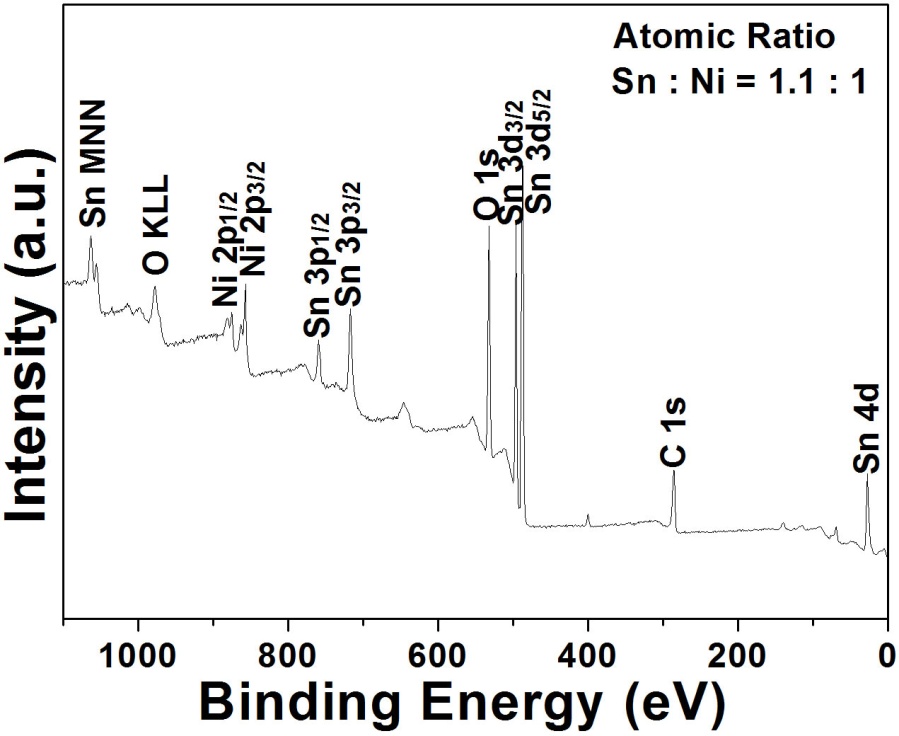


**Figure S7** XPS spectrum of the Sn–Ni/G dual framework.


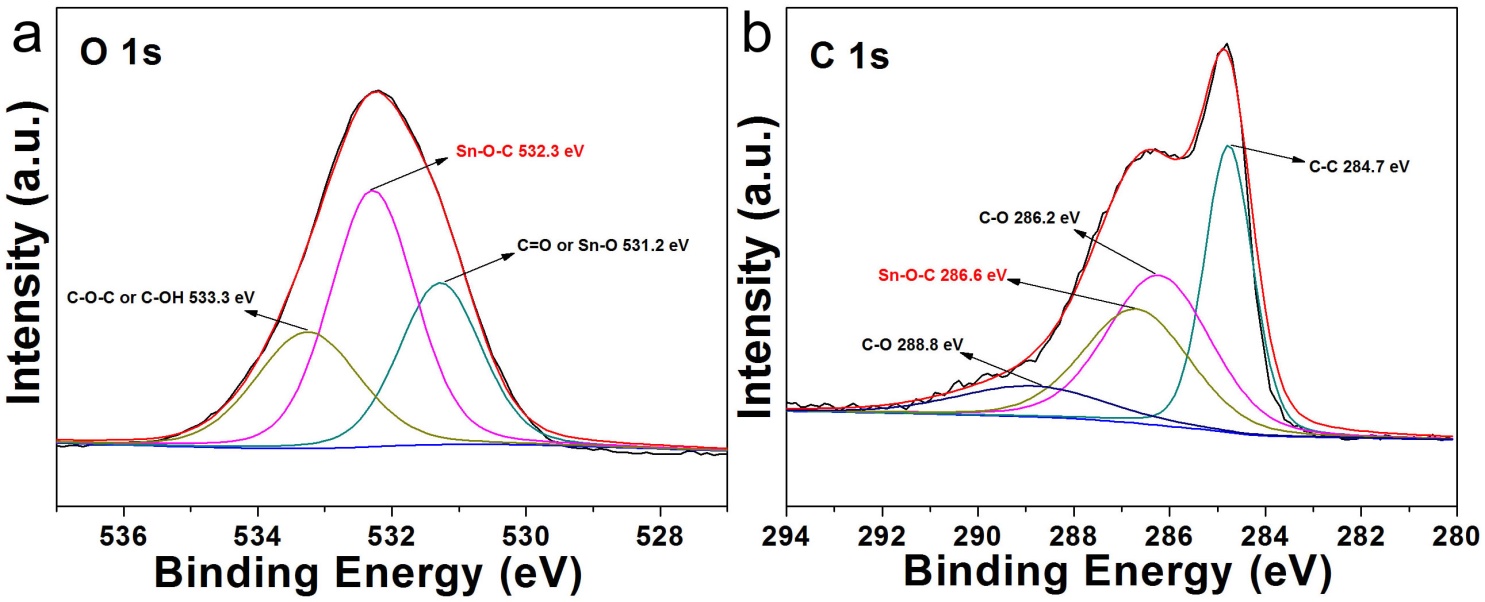


**Figure S8** O 1s and C 1s XPS spectra of the Sn–Ni/G dual framework.

**Table S1** Comparison of the lithium storage performance between the Sn–Ni/G dual framework and previous Sn–M alloy-based anodes.

| **Anode**  **materials** | **Cycling stability**  **(mAh g^-1^)** | **Rate capability**  **(mAh g^-1^)** | **Ref** |
| --- | --- | --- | --- |
| **Sn–Ni/G dual framework** | **701 at 0.1 A g^-1^ (200 cycles)** | **497 at 1 A g^-1^** | **This work** |
| Sn–Fe@C framework | 545 at 0.1 A g^-1^ (200 cycles) | 491 at 1 A g^-1^ | 1 |
| CoSn_2_/α-TiC/C electrode | 479 at 0.1 A g^-1^ (180 cycles) | NA | 2 |
| Ni–Sn annoy anode | 597 at 0.5 C (200 cycles) | NA | 3 |
| meso-Co_0.3_Sn_0.7_ material | 530 at 0.07 A g^-1^ (50 cycles) | ~400 at 1.3 A g^-1^ | 4 |
| Sn–Fe–C composite | 444 at 0.06 A g^-1^ (170 cycles)  430 at 0.6 A g^-1^ (140 cycles) | NA | 5 |
| Sn–Ni@C network | 381 at 0.1 A g^-1^ (100 cycles) | 275 at 1.2 A g^-1^ | 6 |
| Sn–Fe–Co alloy composite | 510 at 0.05 A g^-1^ (50 cycles) | 298 at 1 A g^-1^ | 7 |
| Fe_0.5_Co_0.5_Sn_5_ nanosphere | ~556 at 0.05 C (100 cycles) | NA | 8 |

**References**

1. H. Shi, Z. Fang, X. Zhang, F. Li, Y. Tang, Y. Zhou, P. Wu, G. Yu, *Nano Lett.* **2018**, *18*, 3193-3198.
2. M. G. Park, D. H. Lee, H. Jung, J. H. Choi, C. M. Park, *ACS Nano* **2018**, *12*, 2955-2967.
3. H. Zhang, T. Shi, D. J. Wetzel, R. G. Nuzzo, P. V. Braun, *Adv. Mater.* **2016**, *28*, 742-747.
4. G. O. Park, J. Yoon, J. K. Shon, Y. S. Choi, J. G. Won, S. B. Park, K. H. Kim, W. S. Yoon, J. M. Kim, *Adv. Funct. Mater.* **2016**, *26*, 2800-2808.
5. Z. Dong, R. Zhang, D. Ji, N. A. Chernova, K. Karki, S. Sallis, L. Piper, M. S. Whittingham, *Adv. Sci.* **2016**, *3*, 1500229.
6. H. Shi, A. Zhang, X. Zhang, H. Yin, S. Wang, Y. Tang, Y. Zhou, P. Wu, *Nanoscale* **2018**, *10*, 4962-4968.
7. X. Li, X. He, Y. Xu, L. Huang, J. Li, S. Sun, J. Zhao, *J. Mater. Chem. A* **2015**, *3*, 3794-3800.
8. F. Xin, X. Wang, J. Bai, W. Wen, H. Tian, C. Wang, W. Han, *J. Mater. Chem. A* **2015**, *3*, 7170-7178.
